# Striatal Distribution and Cytoarchitecture of Dopamine Receptor Subtype 1 and 2: Evidence from Double-Labeling Transgenic Mice

**DOI:** 10.3389/fncir.2017.00057

**Published:** 2017-08-17

**Authors:** Keke Ren, Baolin Guo, Chunqiu Dai, Han Yao, Tangna Sun, Xia Liu, Zhantao Bai, Wenting Wang, Shengxi Wu

**Affiliations:** ^1^Department of Neurobiology and Collaborative Innovation Center for Brain Science, School of Basic Medicine, Fourth Military Medical University Xi’an, China; ^2^College of Life Sciences and Research Center for Resource Peptide Drugs, Shaanxi Engineering and Technological Research Center for Conversation and Utilization of Regional Biological Resources, Yanan University Yanan, China; ^3^The Fifth Camp, The First Cadet Brigade, Fourth Military Medical University Xi’an, China; ^4^Department of Neurology, Tangdu Hospital, Fourth Military Medical University Xi’an, China

**Keywords:** striatum, dopamine receptor subtype 1, dopamine receptor subtype 2, medium-sized spiny neurons, BAC transgenic mice

## Abstract

As the main input nucleus of the basal ganglion, the striatum executes different functions, including motivation, reward and attention. The functions of the striatum highly rely on its subregions that receive projections from various cortical areas and the distribution of striatonigral neurons that express D1 dopamine (DA) receptors (or D1 medium-sized spiny neurons, D1 MSNs) and striatopallidal neurons that express D2 DA receptors (or D2 MSNs). Using bacterial artificial chromosome (BAC) transgenic mice, several studies have recently been performed on the spatial distribution of D1 and D2 MSNs. However, these studies mainly focused on enumeration of either D1-enhanced fluorescent protein (eGFP) or D2-eGFP in mice. In the present work, we used Drd1a-tdTamato and Drd2-eGFP double BAC transgenic mice to evaluate the spatial pattern of D1 MSNs (red fluorescence) and D2 MSNs (green fluorescence) along the rostro-caudal axis of the dorsal striatum. The dorsal striatum was divided into three subregions: rostral caudoputamen (CPr), intermediate CP (CPi), and caudal CP (CPc) across the rostral–caudal extent of the striatum. The results demonstrate that D1 and D2 MSNs were intermingled with each other in most of these regions. The cell density of D1 MSNs was slightly higher than D2 MSNs through CPr, CPi, and CPc, though it did not reach significance. However, in CPi, the ratio of D1/D2 in the ventromedial CPi group was significantly higher than those in dorsolateral, dorsomedial, and ventrolateral CPi. There was similar proportion of cells that co-expressed D1 and D2 receptors. Moreover, we demonstrated a pathway-specific activation pattern of D1 MSNs and D2 MSNs in a manic like mouse model induced by D-Amphetamine by utilizing this double transgenic mice and c-fos immunoreactivity. Our results may provide a morphological basis for the function or pathophysiology of striatonigral and striatopallidal neurons with diverse cortical inputs to the dorsal striatum.

## Introduction

The striatum is a cardinal point of the basal ganglia (BG) that plays an important role in action selection, motor planning and other functions related to evaluation and selection (Balleine et al., 2007; Graybiel and Grafton, 2015). A spectrum of movement disorders such as Parkinson’s disease (PD), chorea and dystonia result from diseases of the striatum (Graybiel, 2000; Tanabe et al., 2009; Plotkin and Surmeier, 2015). The striatal projection neurons are GABAergic medium-sized spiny neurons (MSNs) which constitute up to 95% of the neuronal population in the striatum (Kreitzer, 2009). These MSNs are subdivided into two individual populations based on axonal projections. Some belong to the direct pathway that projects axons to the substantia nigra par reticulate (SNr), while the others categorized as the indirect pathway project to the globus pallidus (GP; Kreitzer and Malenka, 2008). These MSNs receive glutamatergic inputs from the cortex and thalamus on one hand, and dopaminergic afferents from the midbrain nigrostriatal that form synapses on its dendrites and spine necks (Gerfen and Surmeier, 2011). The dopaminergic input is critical to normal functioning of the striatum and BG. These two subgroups of MSNs express distinguishing dopamine (DA) receptors. The striatonigral MSNs express DA receptor type 1 (D1); in contrast, striatopallidal MSNs express DA receptor type 2 (D2). D1 and D2 receptors are both G protein-coupled receptors. However, they trigger distinct intracellular signaling pathways and targets. For example, our previous work found that D1 and D2 MSNs received differential dopaminergic regulations of inwardly rectifying potassium channel, and presents a distinct sub-threshold dynamic (Zhao et al., 2016). Thus, D1 and D2 receptors lead to fundamentally different cellular responses to DA information.

A recent work has revealed a meticulous community arrangement in each of the striatum according to the cortico-striatal projectome (Hintiryan et al., 2016). It was reported that the huge spaces occupied by the striatum could be roughly divided into three communities: rostral striatum, intermediate striatum, and caudal striatum according to the region of the striatal main body. Because these areas in each part receive various afferent (Hintiryan et al., 2016), the output and functions diverse greatly. It is possible that functionally diverse areas may represent different proportions of D1 MSNs and D2 MSNs, because of the distinct input and output neuronal circuitry. Although it is widely accepted that D1 MSNs and D2 MSNs are equally expressed in the entire striatum, it still remains unsettled as to the detailed distribution of the two populations in these three areas of the striatum. To address this organizational question of the striatum, visualizing both D1 MSNs and D2 MSNs should be conducted. Because of the bias of antibody and the different effects of double immunochemistry, it is not convenient to visualize the two groups of cells using histochemical methods. Several studies have recently been performed on the spatial distribution of D1 and D2 MSNs using bacterial artificial chromosome (BAC) transgenic mice expressed same eGFP on D1 receptor or D2 receptor (Matamales et al., 2009; Gangarossa et al., 2013). However, it is still not convenient since both D1 MSNs and D2 MSNs mark with same fluorescent protein. Recently, Shuen et al. (2008) developed a BAC mouse line tagged D1 receptor with tdTomato protein, which makes it possible to investigate the spatial pattern of D1 MSNs and D2 MSNs simultaneously. It also will be also useful for exploring the spatial activities’ pattern of both D1 MSNs and D2 MSNs during the functional task or disease-related state.

In the present study, we used a hybrid of the D1-tdTomato and D2-eGFP mouse line in which D1 MSNs and D2 MSNs are labeled simultaneously with red and green fluorescence, respectively. We used it to visualize the D1 and D2 MSNs in 11 areas of the striatum, and investigated the distribution and proportion of these two populations, with the aim of providing morphological evidence for the dopaminergic regulations in different striatal areas. Furthermore, we analyzed the c-fos immunoreactivity in the D1 MSNs and D2 MSNs in an acute manic mouse model induced by intraperitoneal injection of D-Amphetamine (D-AMPH). The results demonstrated a pathway-specific activation of striatal D1 MSNs and D2 MSNs with the cortico-striatal projectome in hyperactivities induced by psychostimulant.

## Materials and Methods

### Animals

The generation of D1-tdTomato and D2-eGFP transgenic mice had previously been described (Gong et al., 2003; Shuen et al., 2008). To label simultaneously D1 and D2 MSNs, we crossed these two mouse lines to obtain double transgenic mice. Adult mice who co-expressed the D1-tdTomato and D2-eGFP were housed in a temperature-controlled environment on a 12-h light/dark cycle (lights on at 08:00 and off at 20:00) with free access to food and water. All experimental procedures were approved by the Institutional Animal Care and Use Committee (IACUC) of the Fourth Military Medical University (Xi’an, China), and carried out according to the “Principles of Medical Laboratory Animal Care” issued by the National Ministry of Health (NIH). All efforts were made to minimize animal suffering and to reduce the number of animals used.

### Drugs and Treatment

D-AMPH (Cayman Chemical, Ann Arbor, MI, USA) was dissolved in saline and administered in a volume of 10 ml/kg. One dose of amphetamine (2 mg/kg body weight) or vehicle was administered by intraperitoneal (i.p.) injection 30 min before placing the mouse into the testing chamber.

### Behavior Test

The animals were allowed to habituate the experimental room at least 12 h before the tests. Mice were received either saline or D-AMPH challenge and placed in the center of a open field chamber (40 cm × 40 cm) for habituating 5 min. Then the locomotor activity was recorded for 30 min. Locomotor activity was analyzed by using the SMART Video Tracking System (Panlab S.L.U.). The chamber was divided into an outer zone (10 cm from the walls) and a square center zone (20 cm × 20 cm). Total distance traveled was used for measuring locomotor activity, and distance traveled in the central zone was used for evaluating anxiety (Gubert et al., 2016; Guo et al., 2017). Two hours after the behavior test, these mice were sacrificed for c-fos immunostaining.

### *In Vitro* Electrophysiological Recording

Mixed gender D1-tdTomato and D2-eGFP mice at 5–8 weeks-old were used for whole-cell electrophysiology procedures. Acute coronal striatal slices were prepared as previous reported (Zhao et al., 2016). Briefly, mice were anesthetized with pentobarbital sodium (30–40 mg/kg body weight) and transcardially perfused with 20 ml of ice-cold carbogenated (95% O_2_, 5% CO_2_) cutting solution containing (in mM): 115 choline-chloride, 2.5 KCl, 1.25 NaH_2_PO_4_, 0.5 CaCl_2_, 8 MgCl_2_, 26 NaHCO_3_, 10 D-(+)-glucose, 0.1 L-ascorbic acid, and 0.4 sodium pyruvate (with osmolarity of 300–305 mOsm). The brains were then rapidly removed and placed in ice-cold cutting solution for slice preparation. The coronal slices (300 μm) were prepared using a slicer (Vibrotome 1000 Plus, Ted Pella Inc., Redding, CA, USA) and then incubated in a holding chamber at 32°C with carbogenated cutting solution for 15–20 min. The slices were then transferred to artificial cerebral spinal fluid (ACSF) containing (mM): 119 NaCl, 2.3 KCl, 1.0 NaH_2_PO_4_, 26 NaHCO_3_, 11 D-(+)-glucose, 1.3 MgCl_2_, 2.5 CaCl_2_ (pH 7.4, with osmolarity of 295–300 mOsm) at room temperature for at least 1 h.

The slices were placed in a recording chamber and constantly perfused with carbogenated ACSF at 24–28°C (TC-324B, Warner Instruments, Hamden, CT, USA). The perfusion rate was 2.0 ml/min. The fluorescently labeled D1 or D2 MSNs were visualized and identified with a microscope equipped with GFP or red fluorescent protein (RFP) filter (BX-51WI, Olympus, Japan). Whole-cell patch clamp recordings were performed with infrared-differential interference contrast (IR-DIC) visualized guide. Recording pipettes (BF150-86-7.5, Sutter Instruments, Novato, CA, USA) were pulled in a horizontal pipette puller (P-97, Sutter Instruments, Novato, CA, USA) with a tip resistance of 3–5 MΩ. Patch pipettes were filled with a solution containing (in mM): 128 potassium gluconate, 10 Hepes, 10 phosphocreatine sodium salt, 1.1 EGTA, 5 ATP magnesium salt, and 0.4 GTP sodium salt. pH was adjusted to 7.3 with KOH, and osmolarity was adjusted to 300–305 mOsm with sucrose. Cells with series resistance more than 20 MΩ at any time during the recordings were discarded. Neurons with resting membrane potentials more negative than −60 mV and action potentials with overshoot were selected for further experiments. Liquid junction potentials were not corrected. An axon 200A amplifier (Molecular Devices, Sunnyvale, CA, USA) was used to record membrane potentials. Signals were low-pass filtered at 5 kHz and sampled at 20 kHz with a Digidata 1322A and Clampex 9.0 (Molecular Devices), and data were stored on a computer for subsequent off-line analysis.

### Cell Filling for Electrophysiologically Characterized Neurons

Some MSNs were labeled by adding 0.5% neurobiotin 350 (SP-1155, Vector laboratories, Burlingame, CA, USA) to the internal solution. During the recording, simple diffusion of the dye from the pipette into the cell was sufficient to obtain complete labeling. Immediately after the recording, the slices were fixed by immersion in 4% paraformaldehyde (PFA), 0.1 M phosphate buffer (PB, pH 7.4) at 4°C. The slices were then cryoprotected by infiltration with 30% sucrose in 0.1 M PB overnight at 4°C, and were incubated at 4°C for 12 h with streptavidin-Alexa Fluor 350 (1:600, S11249, Molecular probe, Carlsbad, CA, USA). The slices were examined under a BX-51 microscope using FV 1200 software (Olympus, Tokyo, Japan). We obtained the labeled cells from 10 to 20 serial optical sections that were 2 μm apart. Using a 3D reconstruction software (Imaris 7.7, Biteplane AG, Zurich, Switzerland), morphology reconstructions were performed from the sets of confocal images.

### Tissue Preparation and Immunohistochemistry

Mice were deeply anesthetized with an overdose of sodium pentobarbital (1%, 40 mg/kg body weight, i.p.) and perfused with 100 ml of 0.01 M phosphate buffered saline (PBS, pH 7.4), followed by 200 ml of 4% PFA in 0.1 M PB. The brains were dissected and post-fixed for 4 h in the same fixative at 4°C, and transferred to 30% (w/v) sucrose for 48 h at 4°C. After that, the brain blocks were cut into 30 μm thick coronal sections on a cryostat. All sections containing dorsal striatum were serially collected into 24 dishes containing 0.01 M PBS, with avoidance from light. Each dish contained a set of serial sections that were stored at 4°C for subsequent immunohistochemical staining.

Coronal striatal sections were washed three times with 0.01 M PBS, blocked with 3% bovine serum albumin (BSA) in 0.01 M PBS at room temperature for 2 h, and then used for immunofluorescent staining. Slices were first incubated at 4°C for 48 h with rabbit anti-D1 receptor antibody (1:100, ab81296, Abcam), goat anti-D2 receptor antibody (1:100, ab32349, Abcam) and rabbit anti-cFos antibody (1:10,000, F7799, Sigma), respectively. The antibody diluent was prepared with 0.01 M PBS containing 0.3% (v/v) TritonX-100, 0.05% (w/v) NaN3, 0.25% (w/v) λ-carrageenan, and 3% BSA. The sections were rinsed three times with 0.01 M PBS and incubated at room temperature for 2 h with Alexa Fluor 647-conjugated goat anti-rabbit IgG (1:500, A-21246, ThermoFisher) and Alexa Fluor 647-conjugated donkey anti-goat IgG (1:500, A-21447, ThermoFisher), respectively. After three times washing, sections were incubated with Hoechst 33342 (1:2000, Sigma) for 15 min at room temperature to stain nuclei. All of the sections were displayed in order with an interval of 180 μm on glass slides. The stained sections were observed and captured under a confocal laser scanning (FV1200, Olympus) and fluorescence (BX-51, Olympus) microscopes.

### Area Division and Cell Counting

After sections containing striatum were taken, the panorama of striatal image was stitched manually by xuvstitch v1.8.0 software (Emmenlauer et al., 2009). Then we used the Imaris software to explore the distribution of D1 and D2 MSNs and c-fos/D1 or c-fos/D2 colocalization in different regions of the striatum. Slices were picked up every 180 μm and divided into continuous coronal slices according to the division methods of striatum used by Hintiryan et al. (2016). Cell counting was achieved by automatic spots algorithm based on the fluorescent threshold using the Imaris software.

### Statistical Analysis

All data were transferred to Prism 6.0 (GraphPad Software, Inc., La Jolla, CA, USA) for analysis and graphing. Data are presented as mean ± SEM, and the “*n*” value given for each experiment refers to the number of slices or cells analyzed. All error bars indicate SEM. Two group results were compared by using an unpaired Student’s *t*-test. Comparisons of more than two groups were analyzed with one-way analysis of variance (ANOVA) or two-way ANOVA. The significance levels for all tests were set at *p < 0.05, **p < 0.01, ***p < 0.001, ****p < 0.0001.

## Results

### D1 and D2 MSNs Marked by D1-tdTomato and D2-eGFP Double Transgenic Mouse

**Figure 1:**
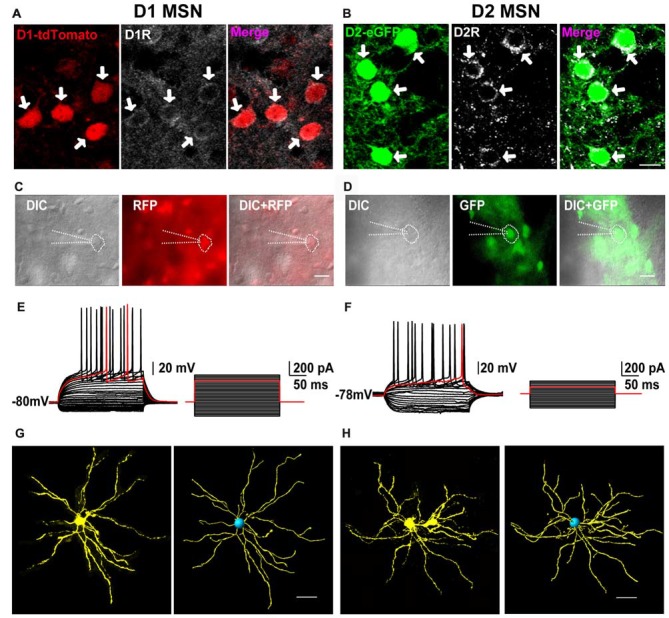
The receptors’ expression, neural firing pattern and dendritic branches of D1-tdTomato and D2-enhanced fluorescent protein (eGFP) positive neurons in the striatum. **(A)** D1 receptor and D1-tdTomato signal were co-expressed in the striatal medium-sized spiny neurons (MSNs). **(B)** D2 receptor and D2-eGFP signal were co-expressed in the MSNs. Scale bar = 10 μm. **(C)** A D1-tdTomato positive neuron was recorded by patch clamp pipette in differential interference contrast (DIC) image mode. Scale bar = 20 μm. **(D)** A D2-eGFP positive neuron was recorded by patch clamp pipette in DIC image mode. Scale bar = 20 μm. **(E)** The same neuron from **(C)** showed typical delay firing pattern in current clamp mode. **(F)** The same neuron from **(D)** also showed typical delay firing pattern and smaller rheobase comparing with the cell in **(E)** in current clamp mode. **(G)** Cell filling with neurobiotin 350 and 3D reconstruction outlined the dendritic branches of a D1-tdTomato positive neuron. Scale bar = 40 μm. **(H)** Cell filling with neurobiotin 350 and 3D reconstruction outlined the dendritic branches of a D2-eGFP positive neuron. Scale bar = 40 μm.

First, we performed immunofluorescent histochemistry to test whether the two fluorescents indeed labeled D1 and D2 receptors in transgenic mice. As shown in Figure 1A, D1 receptor immunoreactivity was co-localized with D1-tdTomato in striatal cells and in red. On the contrary, D2 receptor immunoreactivity was co-expressed with D2-eGFP and in green (Figure 1B). It suggested that D1-tdTomato and D2-eGFP could be used for tagging D1 and D2 receptors, respectively. Next, we wanted to test that these neurons with fluorescent indeed were medium-sized spiny projection neurons (MSNs) of the striatum. We therefore performed whole cell current clamp recordings in the tdTomato- or eGFP-positive neurons (Figures 1C,D). These neurons showed hyperpolarization resting membrane potential (D1: −80.5 ± 1.12 mV, *n* = 14; D2: −81.0 ± 1.19 mV, *n* = 12). With the depolarization current step stimulation, the delayed first action potential was manifest on these neurons (Figures 1E,F). The average delay time of D1 and D2 cells were 168.3 ± 16.37 ms and 179.5 ± 13.80 ms, respectively. It demonstrated that these fluorescent positive cells had typical intrinsic and firing properties of MSNs according to previous reports (Kita et al., 1984; Nisenbaum and Wilson, 1995). Finally, we did cell filling on several recorded cells with neurobiotin to check the morphology of the fluorescent-positive cells. After staining and reconstruction, we found that these neurons had radial dendrites and spines (Figures 1G,H). Taking together, these results suggested that both D1-tdTomato and D2-eGFP could be used for reliable labeling D1 MSNs and D2 MSNs, respectively.

### D1 and D2 MSNs Expression Pattern Across Rostral-Caudal Extent of the Striatum

**Figure 2:**
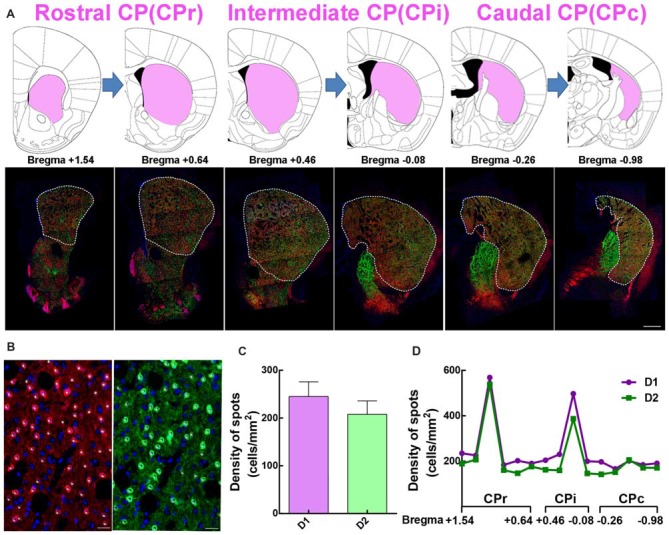
The distribution of the dorsal striatum in different sections and the summary of D1 and D2 MSNs in divided slices. **(A)** The dorsal striatum is composed of rostral caudoputamen (CPr), intermediate (CPi) and caudal (CPc). Upper panel showed half of the picture of coronal slices in Mouse Brain Mapping per 180 μm which contained striatum. Lower panel showed a coronal section of panorama brain sections, which had well correspondence with those slices in upper panel. **(B)** The image showed some white spots which clear overlapped with red D1 MSNs in the left panel and green D2 MSNs in the right panel. The number of white spots was used for quantitative counting the number of D1 MSNs and D2 MSNs based on the fluorescent threshold. Scale bar = 20 μm. **(C)** The value of density of D1 MSNs is slightly bigger than it in D2 MSNs but it did not show significant difference (Density: D1, 245.1 ± 30.75; D2, 207.7 ± 28.16; *n* = 15 slices, *p* > 0.05, unpaired *t*-test). **(D)** A conclusion of the cell density of D1 and D2 MSNs from Bregma +1.54 to Bregma −0.98 per 180 μm in dorsal lateral striatum.

In comparing traditional immunofluorescence to D1 or D2 receptors, the fluorescent signal from transgenic mice is stronger and reliable. We adapted the D1-tdTomato and D2-eGFP double transgenic mouse line as a good choice to analyze the distribution and cytoarchitecture of D1 MSNs and D2 MSNs. First, we dissected rostral CP (CPr) from Bregma +1.54 to +0.64, intermediate CP (CPi) from Bregma +0.46 to −0.08, caudal CP (CPc) from Bregma −0.26 to −0.98 based on Hintiryan et al. (2016) recent work (Figure 2A). Then we performed quantitative analysis of D1 and D2 MSN cell density from CPr to CPc by counting tdTomato or eGFP signal counterstained with Hoechst (Figure 2B). We found that the density of D1 MSNs in whole striatum was slightly higher than that of D2 MSNs, but without statistical difference (Density: D1, 245.1 ± 30.75 cells/mm^2^; D2, 207.7 ± 28.16 cells/mm^2^; *n* = 15 slices, *p* > 0.05, unpaired *t*-test, Figure 2C). Additionally, along the CPr-CPc axis, the density of D1 MSNs and D2 MSNs in different layers showed the same tendency (Figure 2D).

### D1 and D2 MSNs Expression Pattern in Different Regions of the Rostral Striatum

**Figure 3:**
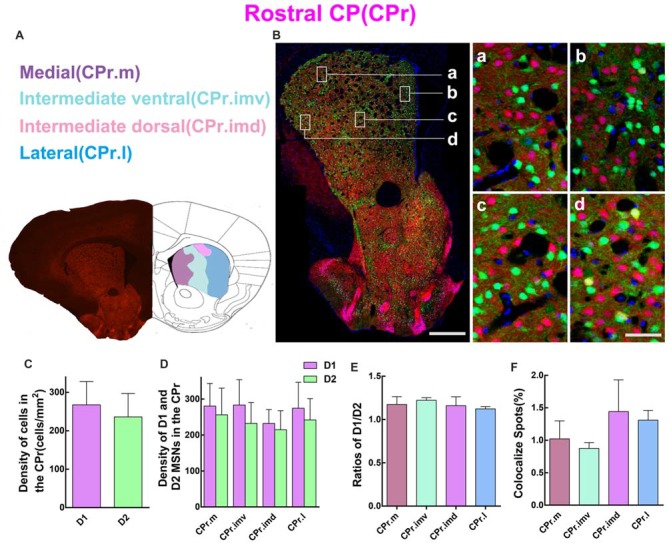
Quantitative analysis of D1 and D2 MSNs in the four subregions of rostral CP. **(A)** Rostral CP was divided into medial (CPr.m), intermediate ventral (CPr.imv), intermediate dorsal (CPr.imd) and lateral (CPr.l). **(B)** The left panel showed a representative section of CPr. The right panel displayed that D1 MSNs and D2 MSNs were almost intermingled with each other in all areas. Scale bar = 400 μm (low-power), Scale bar = 40 μm (high-power in **a–d**). **(C)** The statistics analysis did not reveal significant difference between the cell density in the D1 group and the D2 group in the CPr (Density: D1, 267.6 ± 60.81; D2, 236.2 ± 60.88; *n* = 6 slices, *p* > 0.05, unpaired *t*-test). **(D)** The D1 MSNs and D2 MSNs density (cells/mm^2^) in domains of CPr.m, CPr.imv, CPr.imd and CPr.l did not show significant difference (Density of D1 MSNs: CPr.m, 280.3 ± 60.89; CPr.imv, 283.3 ± 70.54; CPr.imd, 232.0 ± 38.58; CPr.l, 274.5 ± 72.12; Density of D2 MSNs: CPr.m, 256.2 ± 74.01; CPr.imv, 232.5 ± 57.89; CPr.imd, 214.6 ± 52.90; CPr.l, 241.8 ± 59.37; *n* = 6 slices, *p* > 0.05, one-way analysis of variance (ANOVA)). **(E)** The ratios of D1 and D2 MSNs density in the CPr.m, CPr.imv, CPr.imd and CPr.l did not reveal significant difference (Ratio: CPr.m, 1.2 ± 0.09; CPr.imv, 1.2 ± 0.03; CPr.imd, 1.2 ± 0.10; CPr.l, 1.1 ± 0.03; *n* = 6 slices, *p* > 0.05, one-way ANOVA). **(F)** The values of D1 and D2 MSNs co-localized spots in CPr.m, CPr.imv, CPr.imd and CPr.l did not show significant difference (Density of co-localized spots: CPr.m, 1.0 ± 0.28%; CPr.imv, 0.9 ± 0.09%; CPr.imd, 1.4 ± 0.49%; CPr.l, 1.3 ± 0.15%; *n* = 6 slices, *p* > 0.05, one-way ANOVA).

Since striatum from the rostral to caudal received different cortical inputs, it is necessary to perform the quantitative analysis in serial striatal parts. So we first examined the cell density of D1 MSNs and D2 MSNs in CPr. According to a previous study (Hintiryan et al., 2016), the CPr was divided into four areas, including medial CPr (CPr.m), intermediate ventral CPr (CPr.imv), intermediate dorsal CPr (CPr.imd), and lateral CPr (CPr.l). Figure 3A shows the image of brain slice and the division of these four areas. Figure 3B demonstrates the distribution pattern of D1 MSNs (red) and D2 MSNs (green) in a typical layer of CPr. Most green and red cells were found to be intermingled as showed in the right panel of Figure 3B. However, several yellow cells could indeed be found on the high magnification image, which indicates cells expressing both D1 and D2 receptors (Figure 3B, right panel d). We then evaluated the density of D1 and D2 MSNs in CPr. The results indicated that the density of D1 MSNs was similar to D2 MSNs, although the absolute value of D1 was slightly higher (density: D1, 267.6 ± 60.81 cells/mm^2^; D2, 236.2 ± 60.88 cells/mm^2^; *n* = 6 slices, *p* > 0.05, unpaired *t*-test; Figure 3C). Next we compared the density of two types of MSNs in four sub-areas of CPr (Figure 3D). This analysis also showed the similar trend to the whole CPr. The ratios of D1/D2 in these four areas were 1.2 ± 0.09 (CPr.m), 1.20 ± 0.03 (CPr.imv), 1.2 ± 0.10 (CPr.imd), and 1.1 ± 0.03 (CPr.l), respectively (*n* = 6 slices, *p* > 0.05, one-way ANOVA; Figure 3E). Finally we counted the cells expressing both D1 and D2 receptors and found that the proportion of co-localized cells among the total cells in four areas were similar (CPr.m, 1.0 ± 0.28%; CPr.imv, 0.9 ± 0.09%; CPr.imd, 1.4 ± 0.49%; CPr.l, 1.3 ± 0.15%; *n* = 6 slices, *p* > 0.05, one-way ANOVA; Figure 3F).

### D1 and D2 MSNs Expression Pattern in Different Regions of the Intermediate Striatum

**Figure 4:**
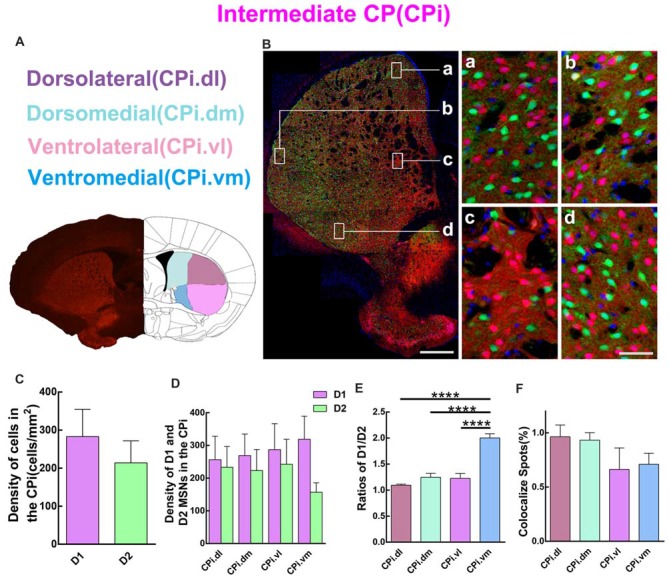
Quantitative analysis of D1 and D2 MSNs in the four areas of intermediate CP. **(A)** Intermediate CP divided into four subregions named dorsolateral (CPi.dl), dorsomedial (CPi.dm), ventrolateral (CPi.vl) and ventromedial (CPi.vm). **(B)** The left panel showed a typical slice of CPi and the right panel displayed that D1 MSNs and D2 MSNs were intermingled with each other in all of four subregions. Scale bar = 400 μm (low-power), Scale bar = 40 μm (high-power in **a–d**). **(C)** The analysis did not show significant difference between the cell density in the D1 group and the D2 group in the CPi (Density: D1, 282.8 ± 71.82; D2, 213.9 ± 57.84; *n* = 4 slices, *p* > 0.05, unpaired *t*-test). **(D)** The D1 MSNs and D2 MSNs density (cells/mm^2^) in areas of CPi.dl, CPi.dm, CPi.vl and CPi.vm did not show significant difference (Density of D1 MSNs: CPi.dl, 256.3 ± 71.76; CPi.dm, 268.9 ± 66.03; CPi.vl, 287.1 ± 79.66; CPi.vm, 318.8 ± 70.54; Density of D2 MSNs: CPi.dl, 233.2 ± 63.54; CPi.dm, 223.2 ± 64.38; CPi.vl, 242.4 ± 76.48; CPi.vm, 156.8 ± 28.58; *n* = 4 slices, *p* > 0.05, one-way ANOVA). **(E)** The ratio of D1 and D2 MSNs density in the CPi.vm group increased significantly compared with groups of CPi.dl, CPi.dm and CPi.vl (Ratio: CPi.dl, 1.1 ± 0.02; CPi.dm, 1.2 ± 0.08; CPi.vl, 1.2 ± 0.10; CPi.vm, 2.0 ± 0.08; *n* = 4 slices, *p* < 0.0001, one-way ANOVA). **(F)** The values of D1 and D2 MSNs co-localized spots in CPi.dl, CPi.dm, CPi.vl and CPi.vm did not reveal significant difference (Density of co-localized spots: CPi.dl, 1.0 ± 0.11%; CPi.dm, 0.9 ± 0.07%; CPi.vl, 0.7 ± 0.20%; CPi.vm, 0.7 ± 0.10%; *n* = 4 slices, *p* > 0.05, one-way ANOVA) ****p < 0.0001.

Quantitative analysis was next performed to assess the spatial distribution of D1 and D2 MSNs in CPi. Similar to CPr, CPi was divided into four sub-regions, including dorsolateral (CPi.dl), dorsomedial (CPi.dm), ventrolateral (CPi.vl) and ventromedial (CPi.vm). The definition of the four areas is shown in Figure 4A. The left panel of Figure 4B shows a panoramic image of CPi and four high magnification images of CPi.dl, CPi.dm, CPi.vl and CPi.vm. The distribution of D1 and D2 MSNs was similar to that in CPr. We then compared the cell density of D1 and D2 MSNs in CPi. The results indicated that the density of D1 MSNs was slightly higher than D2 MSNs but without significant difference (density: D1, 282.8 ± 71.82 cells/mm^2^; D2, 213.9 ± 57.84 cells/mm^2^; *n* = 4 slices, *p* > 0.05, unpaired *t*-test; Figure 4C). Next we counted the density of two types of MSNs in four sub-regions of CPi and found that the absolute value of the density of D1 MSNs was higher than in D2 MSNs, but also this did not reach significance (Figure 4D). We calculated the ratios of D1/D2 in four areas of CPi. Interestingly, the results showed that the ratios of D1/D2 in the CPi.vm group was significantly higher than the CPi.dl, CPi.dm and CPi.vl groups (ratios: CPi.dl, 1.1 ± 0.02; CPi.dm, 1.2 ± 0.08; CPi.vl, 1.2 ± 0.10; CPi.vm, 2.0 ± 0.08; *n* = 4 slices, *p* < 0.0001, one-way ANOVA; Figure 4E). Lastly, we counted the cells expressing both D1 and D2 receptors in CPi. We found that the co-localized spots among total cells presented similar proportions in four areas (CPi.dl, 1.0 ± 0.11%; CPi.dm, 0.9 ± 0.07%; CPi.vl, 0.7 ± 0.2%; CPi.vm, 0.7 ± 0.10%; *n* = 4 slices, *p* > 0.05, one-way ANOVA; Figure 4F).

### D1 and D2 MSNs Expression Pattern in Different Regions of the Caudal Striatum

**Figure 5:**
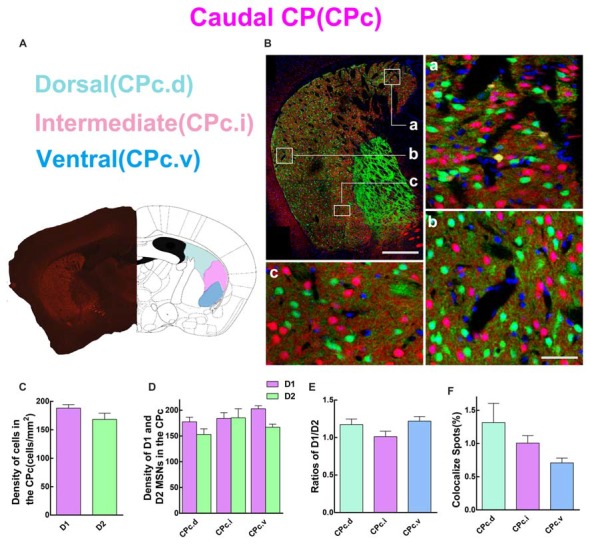
Quantitative analysis of D1 and D2 MSNs in the three subregions of caudal CP. **(A)** Caudal CP was divided into dorsal (CPc.d), intermediate (CPc.i) and ventral (CPc.v). **(B)** The low magnificent image showed a typical slice and three high magnificent images displayed different characteristics of D1 MSNs and D2 MSNs. Scale bar = 400 μm (low-power), Scale bar = 40 μm (high-power in **a–c**). **(C)** The values of MSNs density (cells/mm^2^) in the D1 group and the D2 group did not show significant difference in the CPc (Density: D1, 188.1 ± 6.131; D2, 168.4 ± 10.86; *n* = 5 slices, *p* > 0.05, unpaired *t*-test). **(D)** The D1 MSNs and D2 MSNs density (cells/mm^2^) in areas of CPc.d, CPc.i and CPc.v did not show significant difference (Density of D1 MSNs: CPc.d, 177.4 ± 9.08; CPc.i, 184.0 ± 11.14; CPc.v, 202.8 ± 5.86; Density of D2 MSNs: CPc.d, 152.8 ± 11.01; CPc.i, 185.2 ± 17.58; CPc.v, 167.0 ± 5.83; *n* = 5 slices, *p* > 0.05, one-way ANOVA). **(E)** The ratio of D1 and D2 MSNs density in CPc.d, CPc.i and CPc.v did not show significant difference (Ratio: CPc.d, 1.2 ± 0.07; CPc.i, 1.0 ± 0.07; CPc.v, 1.2 ± 0.06; *n* = 5 slices, *p* > 0.05, one-way ANOVA). **(F)** The values of D1 and D2 MSNs co-localized spots in domains of CPc.d, CPc.i and CPc.v did not show significant difference (Density of co-localized spots: CPc.d, 1.3 ± 0.29%; CPc.i, 1.0 ± 0.11%; CPc.v, 0.7 ± 0.07%; *n* = 5 slices, *p* > 0.05, one-way ANOVA).

Next, we analyzed the spatial distribution of D1 and D2 MSNs in CPc. We separated the CPc into three areas, namely dorsal (CPc.d), intermediate (CPc.i), and ventral (CPc.v). Figure 5A shows the definition of three areas, and Figure 5B demonstrates the distribution pattern of D1 and D2 MSNs in a typical CPc section. The results showed that the cell density of D1 MSNs was also slightly higher than that of D2 MSNs, but without significant difference (density: D1, 188.1 ± 6.131 cells/mm^2^; D2, 168.4 ± 10.86 cells/mm^2^; *n* = 5 slices, *p* > 0.05, unpaired *t*-test; Figure 5C). Figure 5D demonstrates that the cell density of D1 and D2 MSNs were similar in CPc.i. Moreover, the density of D1 MSNs was slightly higher than D2 MSNs in the CPc.d and CPc.v, but did not show significant difference (density of D1 MSNs: CPc.d, 177.4 ± 9.08 cells/mm^2^; CPc.i, 184.0 ± 11.14 cells/mm^2^; CPc.v, 202.8 ± 5.86 cells/mm^2^; Density of D2 MSNs: CPc.d, 152.8 ± 11.01 cells/mm^2^; CPc.i, 185.2 ± 17.58 cells/mm^2^; CPc.v, 167.0 ± 5.83 cells/mm^2^; *n* = 5 slices, *p* > 0.05, one-way ANOVA). This finding was also supported by Figure 5E, which shows the ratios of D1/D2 in the CPc.i were very close. Lastly, the proportion of the co-localized spots of D1 and D2 MSNs was as follows: CPc.d, 1.3 ± 0.29%; CPc.i, 1.0 ± 0.11%; CPc.v, 0.7 ± 0.07% (*n* = 5 slices, *p* > 0.05, one-way ANOVA; Figure 5F).

### D-AMPH-Induced c-fos Expression Pattern in D1 and D2 MSNs Along the CPr-CPc Axis

**Figure 6:**
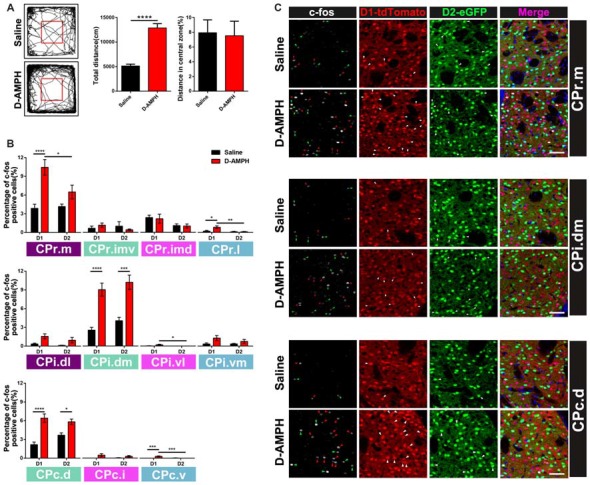
The distribution of c-fos protein expression in D1 or D2 MSNs across the whole CP in the acute manic like state induced by D-Amphetamine (D-AMPH). **(A)** The left panel showed the representative traces of Saline group and D-AMPH group in the open field chamber. The right panel showed the total distance of the mice traveled in D-AMPH group was significant increased compared with saline group (Saline: 5139 ± 366.4 cm, *n* = 10; D-AMPH: 12893 ± 883.1, *n* = 10; *p* < 0.0001, unpaired *t*-test). The distance in central zone did not show difference between Saline group and D-AMPH group (Saline: 7.942 ± 1.748, *n* = 10; D-AMPH: 7.529 ± 1.979, *n* = 10; *p* = 0.88, unpaired *t*-test). **(B)** Upper panel showed the percentage of c-fos positive D1 MSNs or D2 MSNs in the four CPr subregions induced by Saline or D-AMPH (CPr.m: two-way ANOVA with Sidak’s multiple comparisons test, interaction: *p* = 0.02; D1: Saline vs. D-AMPH, *p* < 0.0001; D1:D-AMPH vs. D2:D-AMPH, *p* = 0.03. CPr.l: two-way ANOVA with Sidak’s multiple comparisons test, interaction: *p* = 0.04; D1: Saline vs. D-AMPH, *p* = 0.02; D1:D-AMPH vs. D2:D-AMPH, *p* = 0.006). Medial panel showed the percentage of c-fos positive D1 MSNs or D2 MSNs in the four CPi subregions induced by Saline or D-AMPH (CPi.dm: two-way ANOVA with Sidak’s multiple comparisons test, interaction: *p* = 0.85, saline or D-AMPH treatment: *p* < 0.0001; D1: Saline vs. D-AMPH, *p* < 0.0001; D2:Saline vs. D-AMPH, *p* = 0.0001; CPi.vl: two-way ANOVA with Sidak’s multiple comparisons test, interaction: *p* = 0.09, saline or D-AMPH treatment: *p* = 0.09; D1: D-AMPH vs. D2:D-AMPH, *p* = 0.04). Lower panel showed the percentage of c-fos positive D1 MSNs or D2 MSNs in the three CPc subregions induced by Saline or D-AMPH (CPc.d: two-way ANOVA with Sidak’s multiple comparisons test, interaction: *p* = 0.04; D1: Saline vs. D-AMPH, *p* < 0.0001; D2:Saline vs. D-AMPH, *p* = 0.03; CPc.v: two-way ANOVA with Sidak’s multiple comparisons test, interaction: *p* = 0.09, saline or D-AMPH treatment: *p* = 0.0008; D1: Saline vs. D-AMPH, *p* = 0.0002; D1: D-AMPH vs. D2:D-AMPH, *p* = 0.0002). **(C)** Upper panel showed the representative images displayed c-fos, D1 MSNs and D2 MSNs expression of CPr.m in Saline group and D-AMPH group. Scale bar = 50 μm. Medial panel showed the representative images displayed c-fos, D1 MSNs and D2 MSNs expression of CPi.dm in Saline group and D-AMPH group. Scale bar = 50 μm. Lower panel showed the representative images displayed c-fos, D1 MSNs and D2 MSNs expression of CPc.d in Saline group and D-AMPH group. Scale bar = 50 μm. Green triangle marked D2 MSNs and red triangle marked D1 MSNs. White triangle marked c-fos positive cells. *p < 0.05, **p < 0.01, ***p < 0.001, ****p < 0.0001.

Previous research suggested that D-AMPH could induce acute manic like behavior and trigger the expression of immediate early genes (IEGs) in the striatum (Uslaner et al., 2001; Frey et al., 2006). We did more to investigate the activation pattern of D1 MSNs and D2 MSNs from CPr to CPc in the double transgenic mice with ip injection of D-AMPH by the c-fos immunoreactivity. Indeed the mice of D-AMPH group showed increased total traveled distance but similar central distance percentage in the open field chamber compared with the mice injected with saline (saline group: 5139 ± 366.4 cm; D-AMPH group: 12893 ± 883.1 cm; *p* < 0.0001, unpaired *t* test, Figure 6A). Then we analyzed the percentage of c-fos positive cells in either D1 MSNs or D2 MSNs along the CPr-CPc axis. We found that CPr.m and CPr.l showed increased activities of D1 MSNs in the D-AMPH group when we compared them with D1 MSNs in the saline group or D2 MSNs in the D-AMPH group (two-way ANOVA with Sidak’s multiple comparisons test, interaction: *p* = 0.02; D1: Saline, *n* = 8 slices; D1:D-AMPH, *n* = 7 slices, *p* < 0.0001; D1:D-AMPH vs. D2:D-AMPH, *p* = 0.03, Figure 6B upper panel and 6C upper panel). In the CPi, the mainly activation region was CPi.dm which showed enhanced c-fos expression of D1 MSNs and D2 MSNs in D-AMPH group (two-way ANOVA with Sidak’s multiple comparisons test, D1: Saline, *n* = 8 slices; D1: D-AMPH, *n* = 8 slices, *p* < 0.0001; D2: Saline vs. D2: D-AMPH, *p* = 0.0001, Figure 6B medial panel and 6C medial panel). CPc showed two subregion activations, which were CPc.d and CPc.v. In the CPc.d, the activation pattern was similar with CPi.dm. Both D1 MSNs and D2 MSNs were triggered by D-AMPH (two-way ANOVA with Sidak’s multiple comparisons test, D1: Saline, *n* = 8 slices; D1:D-AMPH, *n* = 8 slices, *p* < 0.0001; D2:Saline vs. D2:D-AMPH, *p* = 0.03, Figure 6B lower panel and 6C lower panel). The activation pattern of CPc.v was similar with CPr.l. D-AMPH induced increased activities in D1 MSNs (two-way ANOVA with Sidak’s multiple comparisons test, D1:Saline, *n* = 8 slices; D1:D-AMPH, *n* = 8 slices, *p* = 0.0002; D2:Saline vs. D2:D-AMPH, *p* = 0.0002, Figure 6B lower panel). Since these subregions of CP received different connections from cortex and other brain areas, our results demonstrated pathway-specific activation of D1 MSNs and D2 MSNs in the striatum.

## Discussion

In this study, we explored the spatial distribution of D1 MSNs and D2 MSNs across the rostral-caudal axis of the striatum using D1-tdTomato and D2-eGFP double transgenic mice. Using these double transgenic mice, two MSN populations were visualized simultaneously, which minimized the experimental variability of traditional immunohistochemical staining. Moreover, we utilized these double transgenic mice and c-fos immunoreactivity to demonstrate a pathway-specific activation pattern of D1 MSNs and D2 MSNs in a manic like mouse model, which provided a potential method to study activation pattern of D1/D2 MSNs balance in striatal related disease.

As the major input nucleus of the BG, the striatum is well known direct and indirect pathways of BG normal function and related diseases. D1 and D2 MSNs critically define these two pathways based on close attention to their cytoarchitecture, spatial distribution, function and distinct modulation. Previous results suggested that D1 and D2 MSNs were intermingled with each other and without clear lamina organization through the whole striatum (Lanca et al., 1986; Gerfen, 1989; Hardman et al., 2002). Thanks to the BAC transgenic mice expressing the fluorescent proteins in D1 or D2 MSNs, we now can easily identify these two cell populations and study their morphological or electrophysiological characteristics (Gong et al., 2003). Previous studies revealed the spatial distribution of D1 and D2 MSNs using either D1-eGFP or D2-eGFP mice (Matamales et al., 2009; Gangarossa et al., 2013). However, they had to analyze D1 and D2 MSNs using different mice since both mouse lines used eGFP tag. It may introduce some variation. In the present work, we studied the spatial distribution patterns of D1 and D2 MSNs simultaneously by taking advantage of the new D1-tdTomato transgenic mice (Shuen et al., 2008). With D1-tdTomato and D2-eGFP double transgenic mice, we can clearly demonstrate the admixture of D1 and D2 MSNs in the same mouse, which is consistent with previous reports (Matamales et al., 2009; Gangarossa et al., 2013). It also simplifies the whole experimental procedure. We also can do serial studies on the spatial information processing of D1 and D2 MSNs simultaneously by using this mouse line. It may provide more information for the balance between the direct and indirect striatal pathways in the future.

The whole striatal regions are organized into distinct input-output sub-networks with functionally diverse D1 and D2 principal neurons located in a unique manner in the striatum (Gerfen, 1989; Gerfen et al., 1990). We divided the striatum into 11 regions according to their different cortical inputs and important roles in brain functions (Hintiryan et al., 2016). We found that D1 and D2 MSNs presented relative balanced expressing characteristics in most regions of the striatum with slightly higher intensity in D1 MSNs, which was consistent with the findings using D1-eGFP and D2-eGFP mice (Matamales et al., 2009; Gangarossa et al., 2013). However, the ventromedial intermediate CP presented a significantly higher ratio of D1/D2 MSNs, which was different from other regions (Figure 4E). The cortical afferents CPi.vm receives are mainly from insular cortex, auditory cortex, perirhinal area, piriform cortex, visual area and secondary motor area. Majercikova et al. (2014) demonstrated that this region is activated under chronic unpredictable variable mild stress. Atallah et al. (2014) found that ventromedial striatum played a critical role in both affect and reinforcement learning. According to the behavioral evidence, high expressing D1 receptor in CPi.vm may indicate an enhancement in behavior reinforcement of physiological status, which is an important question worthy of further study in the future.

The balance of the D1 MSNs and the D2 MSNs are important for the normal function of the striatum (Yin et al., 2009; Aceves et al., 2011). The imbalances of these two pathways were found in such as Parkinson diseases, dyskinesia and addiction (Kravitz et al., 2010; Lobo et al., 2010; Bagetta et al., 2012). Previous reports suggested that D-AMPH could induce manic like behavior and increase DA releasing in the striatum (Frey et al., 2006; Goodwin et al., 2009). And D-AMPH could increase the c-fos mRNA of both striatal D1 MSNs and D2 MSNs in a novel environment (Uslaner et al., 2001). In the present research, we found D-AMPH induced a specific spatial c-fos expression in D1 MSNs and D2 MSNs and also imbalanced activation of these two populations along the whole CPr-CPc axis. The activation of subregions included CPr.m, CPr.l, CPi.dm, CPi.vl, CPc.d and CPc.v. The activities of D1 MSNs in CPr.m, CPr.l, CPi.vl, and CPc.v were higher than D2 MSNs after D-AMPH injection. Recent research suggested that the CPr.l and CPi.vl integrated the somatic sensorimotor and synchronized motor actions (Hintiryan et al., 2016). So Enhanced D1/D2 MSNs c-fos ratio in the CPr.l and CPi.vl could facilitate the direct pathway output and induced the hyperactivities. For the CPc.v and CPr.m, they mainly received the cortical afferent which were involved in autonomic function and emotion. The imbalance of D1 MSNs and D2 MSNs in these two regions might be explained by the psychostimulant role of DA induced by D-AMPH. The mouse cortico-striatal projectome demonstrated that a narrow strip from CPr.m to CPi.dm to CPc.d received the axonal projections from visual and auditory inputs and temporal association areas with perception and also integrated the information for attention and decision making (Hintiryan et al., 2016). D-AMPH induced a significant increasing in the activities of both D1 MSNs and D2 MSNs in CPi.dm and CPc.d. It suggested that these regions might be not only modulated by DA but also other neurotransmitters. Taking together, our results demonstrated a spatial profile of D1 MSNs and D2 MSNs in manic like behavior, which might provide the information for understanding the role of direct pathway and the indirect pathway in normal function and related diseases.

The striatum can also be divided into striatal micro-zones, including striosomes and matrix according to differential expression of neurotransmitter-related molecules (Graybiel, 1984; Gerfen, 1989). Current studies have demonstrated that the matrix is mainly involved in selection behavior related circuits while striosomes is related to evaluation process (Stephenson-Jones et al., 2016). Moreover, D1 and D2 MSNs play different roles in functional inputs and outputs in the matrix and striosomes, by receiving afferent cortical information and by passing information to different brain areas (Friedman et al., 2015). In the matrix, D1 MSNs contributes to “GO” and D2 MSNs contributes to “NO GO”; however, in striosomes, D1 MSNs receive and process “Reward” information and on the contrary, “Punishment” is associated with D2 MSN functions. To some degree, the distribution and proportion D1 and D2 MSNs may indicate circuitry dominant evidence in different striatal areas. A recent work showed that dysfunctional GABA signaling of matrix MSNs might produce motor behavioral changes similar to symptoms of Huntington’s disease (Reinius et al., 2015). It may be interesting to perform quantitative analysis of D1 s and D2 MSNs in striosomes and matrix to provide more information of striosomes and matrix related function.

In this study, we used a double labeling transgenic mouse line to simultaneously visualize D1 and D2 MSNs, and found intermingled distribution patterns across the rostral-caudal extent of the striatum, and that the density of D1 MSNs was slightly higher than that of D2 MSNs. Besides, we found a specific spatial pattern of D1 MSNs and D2 MSNs activation in a manic like behavior, which provided an evidence for the unique function of the striatum.

## Author Contributions

WW and SW conceived the project and designed the experiments. KR and BG performed the experiments, and analyzed the data. CD, HY and TS analyzed the data. WW and SW prepared the manuscript based upon the draft by KR, and BG, XL and ZB revised it critically for important intellectual content. All authors approved the final version of the manuscript submitted for publication, all persons designated as authors qualify for authorship, and all those who qualify for authorship are listed.

## Conflict of Interest Statement

The authors declare that the research was conducted in the absence of any commercial or financial relationships that could be construed as a potential conflict of interest.
